# Anti-proliferative potential and oxidative reactivity of thermo-oxidative degradation products of stigmasterol and stigmasteryl esters for human intestinal cells

**DOI:** 10.1038/s41598-023-34335-0

**Published:** 2023-05-01

**Authors:** Maria Kasprzak, Magdalena Rudzińska, Wojciech Juzwa, Anna Olejnik

**Affiliations:** 1 Department of Biotechnology and Food Microbiology, Poznan University of Life Sciences, 48 Wojska Polskiego St., 60-627 Poznań, Poland; 2 Institute of Food Technology of Plant Origin, Faculty of Food Science and Nutrition, Poznan University of Life Sciences, 31 Wojska Polskiego St., 60-624 Poznań, Poland

**Keywords:** Nutrition, Sterols

## Abstract

Stigmasterol in free and esterified form is incorporated in LDL cholesterol-lowering food products, intended for direct consumption and cooking, baking, and frying. Under thermal treatment, stigmasterol compounds may constitute a source of thermo-oxidative degradation products and oxyderivatives with potentially adverse health effects. This study aimed to analyze the anti-proliferative potential and genotoxicity of thermo-oxidatively treated stigmasterol (ST), stigmasteryl linoleate (ST-LA), and oleate (ST-OA). The effects on cell viability and proliferation, cell cycle progression, intracellular reactive oxygen species (ROS) generation, and DNA damage were analyzed in normal human intestinal cells. The mutagenic potential was assessed in a bacterial reverse mutation test using *Salmonella enterica* serovar Typhimurium strains involving metabolic activation. Stigmasteryl esters showed a significantly lower potential to affect intestinal cell viability and proliferation than non-esterified ST, regardless of heating. Thermo-oxidatively treated ST suppressed intestinal cell proliferation by arresting the cell cycle in the G_2_/M phase and DNA synthesis inhibition. The enhanced intracellular ROS generation and caspase 3/7 activity suggest targeting intestinal cells to the apoptosis pathway. Also, heated ST-LA intensified ROS production and elicited pro-apoptotic effects. Thermo-oxidative derivatives of ST and ST-LA may evoke harmful gastrointestinal effects due to their high oxidative reactivity towards intestinal cells.

## Introduction

Phytosterols are found in various natural sources, including vegetable oils, nuts, grains, vegetables, and fruits^1^. They are also widely incorporated into multiple groups of foodstuffs as biologically active ingredients with a well-documented potential for lowering high serum LDL cholesterol levels. Thus, in recent years, dietary exposure to phytosterols has been significantly increased to a relatively high intake, reaching about 3 g/day^2^.

Generally, phytosterols and their esters are considered safe for humans^2,3^; however, possible adverse effects related to phytosterol intake have been reported in several in vitro and in vivo studies^1,4^. Harmful health effects of phytosterols may be associated with their autoxidation occurring inside and outside the human body and leading to the formation of phytosterol oxidation products (POPs), named oxyphytosterols^5,6^. The most common autoxidative degradation pathway occurs via free radical mechanisms mediated by reactive oxygen species (ROS)^7^. The oxidation process undergoes more rapidly during food storage and thermal treatment, and the generation of phytosterol oxyderivatives is significantly enhanced^8,9^.

POPs, likewise, cholesterol oxidation products (COPs), are commonly identified in human plasma and tissues^10–12^, raising concerns about their safety and possible adverse health effects, including cytotoxicity and pro-atherogenicity. Luister et al.^12^ observed that patients with coronary artery disease with severe aortic stenosis are characterized by increased concentration of plant sterols in plasma and their deposition in aortic valve tissue. Similarly, the elevated plasma POPs concentrations were found in impaired glucose tolerance or type 2 diabetes patients, which may suggest a link between POPs and health status^13^. Furthermore, animal studies showed that a high-fat diet enriched with POPs increased the severity of atherosclerotic lesions^14^. Some evidence also documented that POPs can affect endothelial cells. For example, oxysitosterols have been found to increase the ROS level in rat aortic endothelial cells, enhance cyclooxygenase 2 (Cox-2) expression, and attenuate vasorelaxation in intact endothelium^15^. Another research reported that β-sitosterol epoxy-derivatives inhibited human abdominal aorta endothelial cell growth, but their inhibitory potential was significantly lower than mater compound. Similarly, the POP fraction derived from heated rapeseed oil slightly decreased endothelial cell viability. In contrast to β-sitosterol, its epoxy-derivatives did not induce apoptosis in human endothelial cells^16^. Several potential mechanisms have been proposed through which POPs may promote the development of atherosclerosis, including modulation of lipid homeostasis, cell death triggering, and activation of the oxidation process and inflammation. POPs are suspected of pro-inflammatory activity; however, few scientific reports with significantly differing results do not provide convincing evidence. 7-keto-stigmasterol was found to elicit an inflammatory response in colon cancer Caco-2 cells; it increased pro-inflammatory cytokines TNF-α and IL-8 and anti-inflammatory cytokine IL-10^17^. In contrast, 7β-hydroxysitosterol and 7-ketositosterol did not induce an immune response in human monocytic U937 cells^18^.

The adverse properties of POPs may also result from their cytotoxicity, which depends on phytosterol, the oxidation process, and the cell type and origin^19^. Ryan et al.^20^ reported cytotoxicity of oxysitosterol mixture to the U937 monocytes, colon cancer Caco-2 cells, and hepatoma HepG2 cells, with apoptosis induction only in the U937 cells^20^. Individual oxystigmasterols (7β-OH, epoxydiol, diepoxide) also induced apoptosis in U937 cells with down-regulation of the anti-apoptotic pro-survival Bcl-2 protein^21^. 7-Ketophytosterol oxides, including 7-ketositosterol, 7-ketocampesterol, 7-ketobrassicasterol, and 7-ketostigmasterol suppressed human intestinal carcinoma cell proliferation. 7-ketositosterol and 7-ketocampesterol were characterized by the highest inhibitory potential related to cell cycle arrest and apoptosis induction with increasing caspase-3 activity and down-regulating Bcl-2. 7-ketobrassicasterol also displayed pro-apoptotic activity contrary to 7-ketostigmasterol^22^. Similarly, 7-ketostigmasterol did not induce apoptosis in colon cancer Caco-2 cells, and even it reduced 7-ketocholesterol cytotoxic effects^23^. Knowledge about the cytotoxicity and atherogenicity of POPs and their potential inflammatory effects in humans are still insufficient and contradictory in some cases. Many reports postulate the need for further in vitro and in vivo studies to prove phytosterol safety and determine recommendations for the storage and heat treatment of functional foods enriched with phytosterols^4,19^.

Stigmasterol (ST), one of the most abundant sterols in plants, besides β-sitosterol and campesterol, highly contributes to the human diet. This phytosterol is gaining increasing interest from functional foods manufacturers and consumers for its health-promoting properties, including antihypercholesterolemic, anticancer, anti-osteoarthritis, anti-inflammatory, antidiabetic, immunomodulatory, antiparasitic, antifungal, antibacterial, antiviral, antioxidant, and neuroprotective properties, which are discussed in a recent review article by Bakrim et al.^24^. However, low solubility in oil and high melting point limit ST bioavailability and practical use in the food industry. Functional and applicable properties of ST can be significantly improved by chemical or/and physical modifications, including mainly the esterification with medium or long-chain fatty acids and microencapsulation. The esterification with oleic, linoleic, linolenic, and acetic acids is considered an efficient strategy for increasing the total content of phytosterol in foods and its solubility in oil^4^.

Previously reported studies showed that during thermo-oxidative treatment of ST and its esters with oleic acid (ST-OA) and linoleic acid (ST-LA), a diverse group of derivatives is formed, and their contribution significantly depends on the temperature and exposure time. Free ST was found to degrade faster than its esters generating higher amounts of degradation products, including ST oxides (7α-hydroxy-ST, 7β-hydroxy-ST, 5α,6α-epoxy-ST, 5β,6β-epoxy-ST, stigmasten-3β,5α,6β-triol, and 7keto-ST), non-polar and polar dimers, trimers, and other oligomers^25^. Moreover, thermo-oxidative derivatives formation is also affected by the chemical structure of lipids incorporated into molecules^26^. The protective effect of the fatty acid moiety was also documented; therefore, stigmasteryl esters incorporation into sterol-enriched products to maintain their health-promoting properties and ensure their safety during cooking and processing was postulated^6,25,27^. Preliminary cytotoxicity experiments suggested the higher cytotoxic potential of free ST than its esters to the normal human cells derived from the small intestine, colon mucosa, and liver^6,25^. However, more extensive research is required on the cytotoxicity and health safety of ST and its esters, especially their degradation products and oxyderivatives resulting from thermo-oxidative treatment.

**Figure 1:**
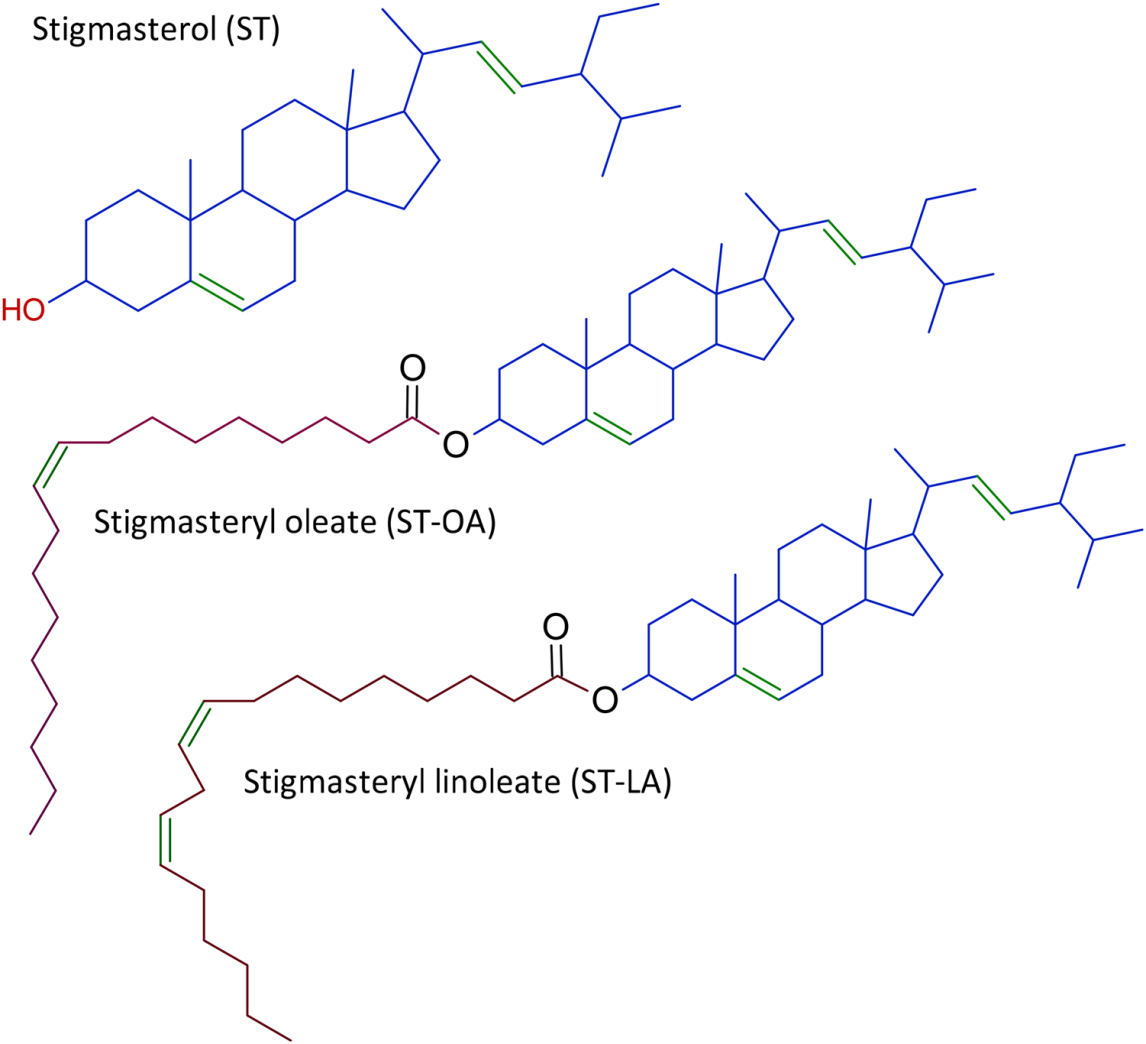
Structure of stigmasterol (ST) and its esters with oleic (ST-OA) and linoleic (ST-LA) acid.

This study aimed to assess the cytotoxic, genotoxic, and mutagenic potential of free ST and linoleic and oleic stigmasteryl esters (Fig. 1) subjected to thermal treatment at 180 °C for 8 h in an oxygen atmosphere. The effects on cell viability, proliferation, DNA synthesis, cell cycle progression, and DNA damage were analyzed in normal human colon mucosa cells. Considering that most phytosterols (> 95%) are not absorbed in the gastrointestinal tract and enter the colon intact^3,28^, the employment of colonic epithelial cells in ST cytotoxicity and genotoxicity experiments is highly justified. Combining data from this study with previously published results of identification analysis of thermo-oxidative derivatives^25^ will provide a more comprehensive overview of how ST and stigmasteryl esters subjected to thermal treatment can affect human gastrointestinal cells.

## Results

### Cytotoxicity of stigmasterol and its esters to normal colon mucosa cells

**Figure 2:**
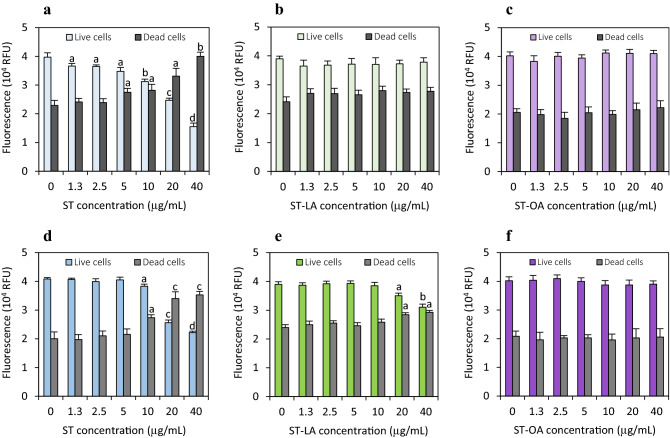
Effect of stigmasterol (ST) (**a**,**d**), stigmasteryl linoleate (ST-LA) (**b**,**e**), and stigmasteryl oleate (ST-OA) (**c**,**f**) non-heated (**a**–**c**) and heated at 180 °C for 8 h (**d**–**f**) on the colon CCD 841 CoN cell viability after 48-h treatment with the analyzed compounds at concentrations ranging from 1.25 to 40 μg/mL. Cell viability (live cell fluorescence) and cytotoxicity (dead cell fluorescence) were determined using MultiTox-Fluor Multiplex Cytotoxicity Assay. The values represent the means (n = 3) ± SD. ^a^*P* < 0.05, ^b^*P* < 0.01, ^c^*P* < 0.001, ^d^*P* < 0.0001 relative to vehicle-treated control cells (Tukey’s post hoc test).

The cytotoxic effects of ST and its esters (ST-LA and ST-OA) (Fig. 1) non-heated and heated at 180 °C for 8 h on human normal colon mucosa CCD 841 CoN cells were determined after a 48-h treatment using the MultiTox-Fluor Multiplex Cytotoxicity Assay. As shown in Fig. 2a, ST induced a dose-dependent decrease in cell viability, which correlated with an increase in the number of dead cells. The non-heated ST at the maximum dose tested (40 μg/mL) reduced the number of viable cells by approximately 80%. The first cytotoxic effects were observed in the colon cells treated with a low ST concentration of 1 μg/mL. Following the heating process, ST evoked significantly less effect on cell proliferation and viability. The first signs of ST cytotoxicity were observed at doses higher than 5 μg/mL. Although, the heated ST at a concentration of 40 μg/mL induced pronounced cytotoxic effects leading to an increase in the dead cell number by 74% (Fig. 2d).

Unlike ST, its non-heated ST-OA and ST-LA esters at doses up to 40 μg/mL did not affect colon cell viability (Fig. 2b,c). Upon the treatment with heated ST-LA at doses of 20 and 40 μg/mL, a reduction in cell viability correlated to an increase in the number of dead cells was observed. In the exposed cell cultures, ST-LA reduced relative cell viability by 10% and 22%, respectively (Fig. 2e). In contrast, the thermal process did not significantly increase ST-OA cytotoxicity (Fig. 2f).

### The effect of stigmasterol and its esters on DNA synthesis in normal colon mucosa cells

**Figure 3:**
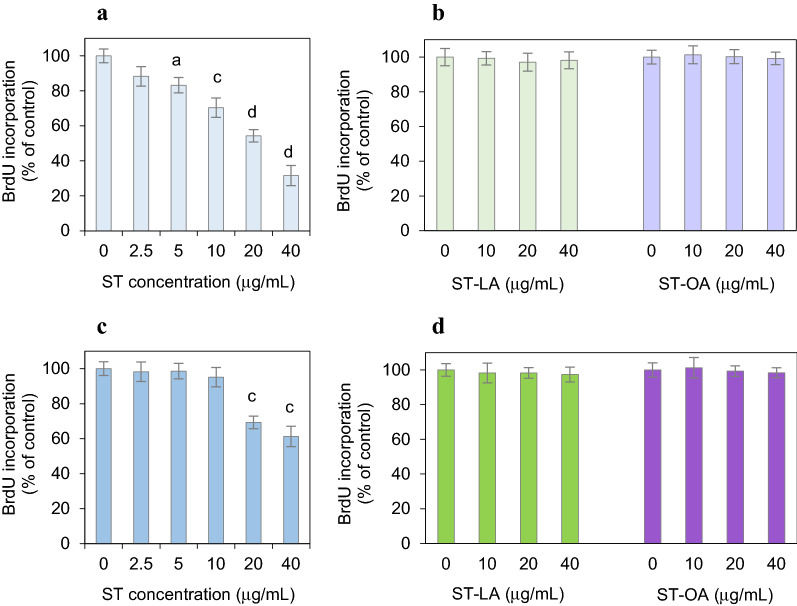
Dose-dependent inhibition of DNA synthesis in the colon mucosa CCD 841 CoN cells by stigmasterol (ST) (**a**) and stigmasteryl esters (ST-LA, ST-OA) (**b**) non-heated (**a**,**b**) and heated at 180 °C for 8 h (**c**,**d**). Values represent the means ± SD (n = 3). ^a^*P* < 0.05, ^b^*P* < 0.01, ^c^*P* < 0.001, ^d^*P* < 0.0001 relative to vehicle-treated control cells (Tukey’s post hoc test).

The treatment of the CCD841 CoN cells with ST affected DNA synthesis substantially. The amount of BrdU incorporated into newly synthesized DNA decreased dose-dependently in the cells treated with non-heated ST (Fig. 3a). ST at 5 μg/mL concentration was the first dose that significantly inhibited DNA synthesis (↓10%, *P* = 0.028). ST at the maximum concentration tested (40 μg/mL) decreased DNA synthesis by 61% (Fig. 3a). ST thermal treatment lowered ST inhibitory potential. The heated ST disturbed DNA synthesis when the highest concentrations of 20 μg/mL and 40 μg/mL were applied, reducing BrdU incorporation in newly synthesized DNA by 31% and 39% at these doses (Fig. 3c). DNA synthesis inhibition was not observed after treating normal colon mucosa cells with esters ST-LA and ST-OA, regardless of their thermal processing (Fig. 3b,d).

### Effect of stigmasterol and its esters on cell cycle progression and apoptosis

**Figure 4:**
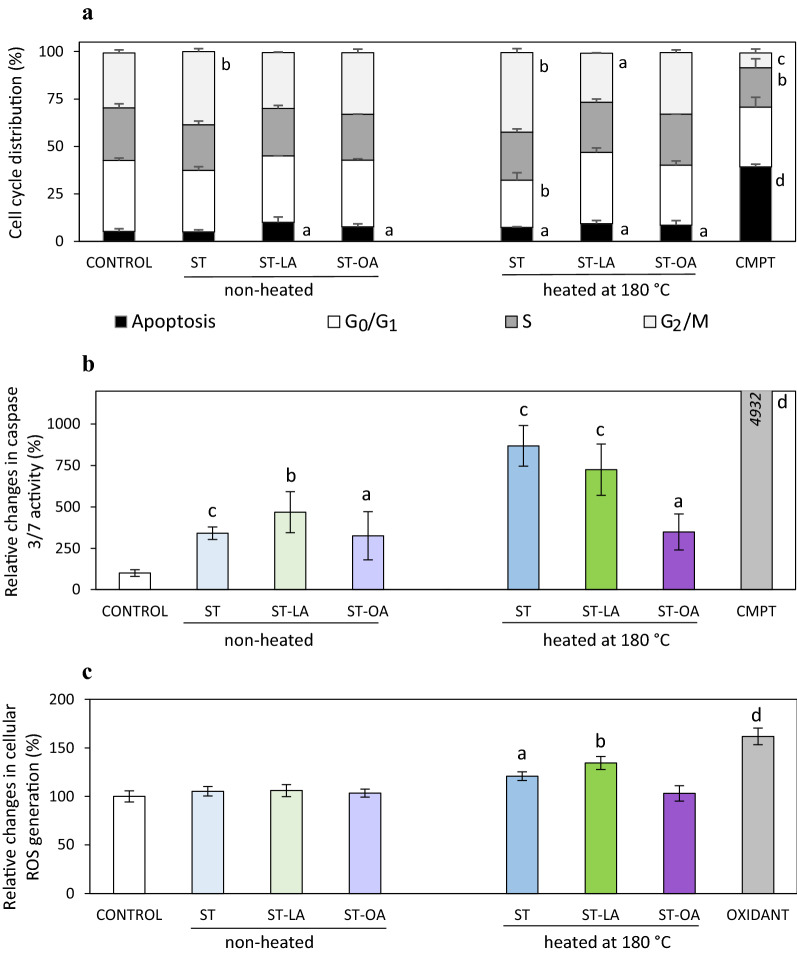
Cell distribution in different cell cycle phases (**a**), caspase 3/7/ activity (**b**), and intracellular reactive oxygen species (ROS) generation (**c**) in the colon mucosa CCD 841 CoN cells treated with non-heated and heated (180 °C, 8 h) stigmasterol (ST), stigmasteryl linoleate (ST-LA) and stigmasteryl oleate (ST-OA) at a concentration of 40 μg/mL. Camptothecin (CMPT, 50 nM) and H_2_O_2_ (OXIDANT, 50 μM) were positive controls. Values represent the means ± SD (n = 3). ^a^*P* < 0.05, ^b^*P* < 0.01, ^c^*P* < 0.001, ^d^*P* < 0.0001 relative to vehicle-treated control cells (Student’s *t*-test).

To investigate how ST, ST-LA, ST-OA, and their thermal degradation products can influence the growth of human normal colon mucosa CCD841 CoN cells, the cell cycle was analyzed by flow cytometry. Cell distribution in different cell cycle phases is presented in Fig. 4a. The results indicate that the compounds at the maximum dose analyzed (40 μg/mL) interfered with the progression of the normal cell cycle of CCD841 CoN cells. The non-heated and heated ST induced significant cell accumulation in the G_2_/M phases. Moreover, ST subjected to the thermal process caused an increase in the subpopulation of dead cells with lower DNA content and decreased the G_0_/G_1_ phase cell population. Slight expanding the cell subpopulation with reduced DNA content was also observed in the cultures treated with stigmasteryl esters (ST-LA and ST-OA) at the maximum dose of 40 μg/mL, indicating their cell death-inducing effect, regardless of heating (Fig. 4a).

Moreover, treatment with test compounds induced caspase-3/7 activation in colon mucosa cells. The highest activation of caspase-3/7 was observed in cells exposed to ST (8.7-fold) and ST-LA (7.3-fold) after heat treatment (Fig. 4b). Also, exposure of cells to heated ST and ST-LA enhanced intracellular ROS production, which increased by 21% and 34%, respectively (Fig. 4c).

### Effect of stigmasterol and its esters on DNA damage in normal colon mucosa cells

**Figure 5:**
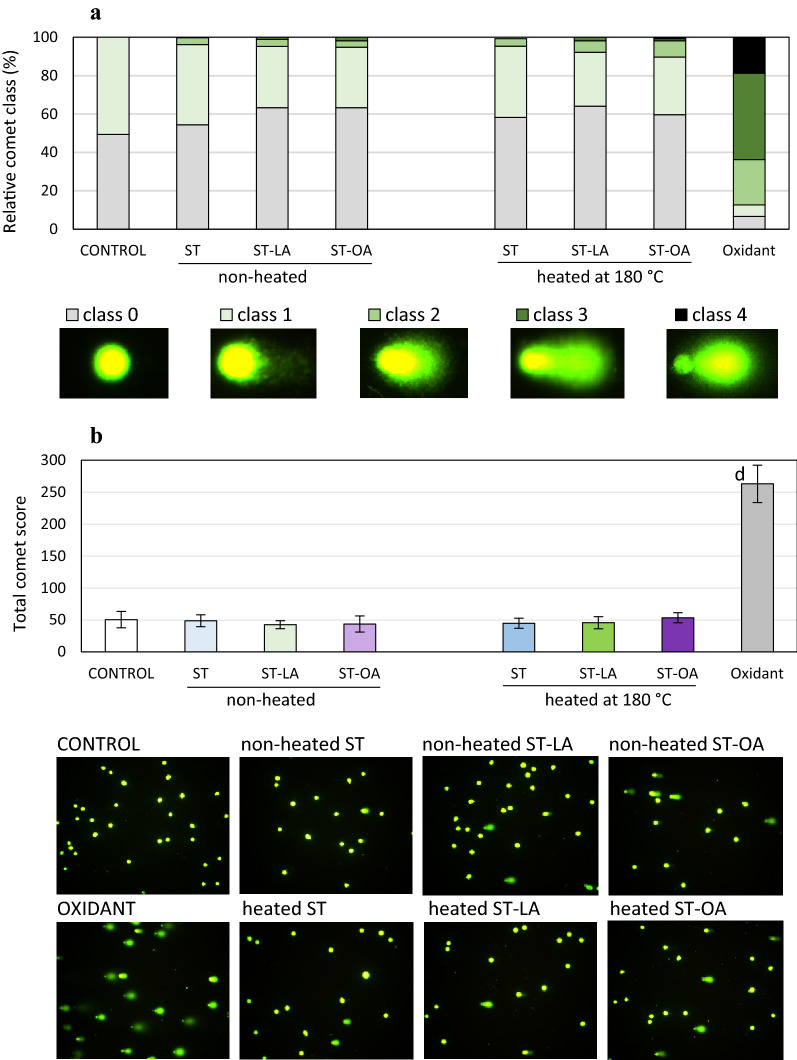
DNA strand breaks in the colon mucosa CCD 841 CoN cells induced by stigmasterol (ST) and its esters (stigmasteryl linoleate, ST-LA, and stigmasteryl oleate, ST-OA) non-heated and heated at 180 °C for 8 h. All compounds were applied at a dose of 40 μg/mL. An oxidant (H_2_O_2_, 100 μM) was used as a positive control in the experiments. Based on the DNA content in the tails, comets were classified into 5 categories: class 0 (no damage), < 1%; class 1 (low damage), 1–25%; class 2 (medium damage), > 25–45%; class 3 (high damage), > 45–70%; class 4 (very high damage), > 70%. Comet class distribution is presented in figure (**a**). Total comet score (TCS), calculated as TCS = 0(n) + 1(n) + 2(n) + 3(n) + 4(n), where “n” means the number of cells in each comet class, is shown in figure (**b**). Values represent the means ± SD (n = 3). ^a^*P* < 0.05, ^b^*P* < 0.01, ^c^*P* < 0.001, ^d^*P* < 0.0001 relative to vehicle-treated control cells (Student’s *t*-test). Photos present CCD 841 CoN cells treated with non-heated and heated ST, ST-LA, and ST-OA at 40 μg/mL concentration and analyzed in the comet assay. Photos were taken at a magnification of 100 ×.

Data obtained from comet assay indicates that ST and its esters at the highest concentration (40 μg/mL) did not induce extensive DNA strand breaks in the colon mucosa cells. Slight DNA damage was shown by classifying comets according to the range of % DNA in the tail (Fig. 5a). Only two comet classes: no damage and low damage (class 0 and 1), were identified in the control cell population. Cell treatment with non-heated or heated ST resulted in the appearance of comets category 2 characterized by medium DNA damage with DNA content in the tails ranging from 25 to 45%. The comets category 2 constituted 3.5% and 3.8% of the populations treated with non-heated or heated ST, respectively. Also, ST-LA and ST-OA caused medium DNA damage, mainly when thermally processed esters were applied. Cell populations with medium DNA damage were estimated at 8.5% and 5.9%, respectively, following treatment with ST-LA and ST-OA (Fig. 5a). Moreover, colon mucosa cells with high DNA damage (DNA content of 45–70% in comet tails) accounted for 1–2% of the cell population exposed to ST esters, regardless of their heat treatment. Although few comets with high damage DNA content were observed, the total comet score (TCS) showed no significant differences in the comet class distribution after cell exposition to both non-heated and heated ST, ST-LA, and ST-OA. Very high DNA damage (> 70% DNA in comet tail) was identified only in cells exposed to 100 μM H_2_O_2_ used as reference oxidant (Fig. 5b), which was reflected in high TCS value (263.1 ± 29.4) (Fig. 5b).

### Mutagenic activity of stigmasterol and its esters

**Figure 6:**
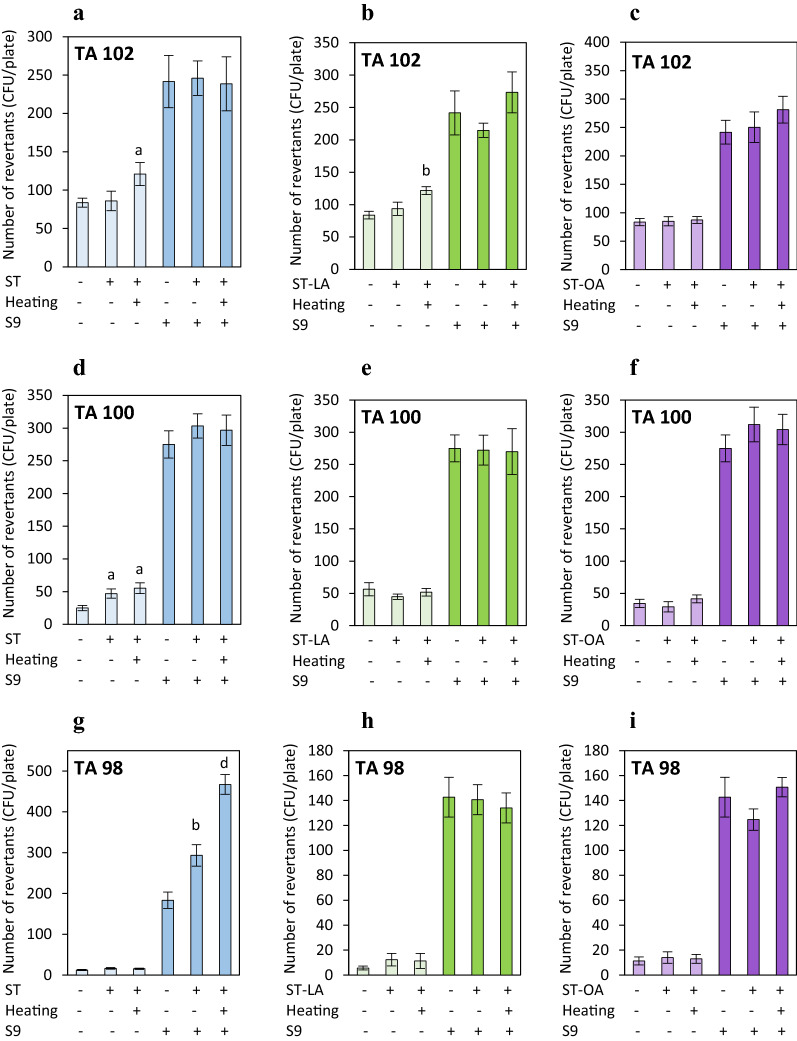
The mutagenic potential of stigmasterol (ST), stigmasteryl linoleate (ST-LA), and stigmasteryl oleate (ST-OA) non-heated and heated at 180 °C for 8 h, applied at a dose of 40 μg/mL in the treatment of three *Salmonella typhimurium* TA102 (**a**–**c**), TA100 (**d**–**f**) and TA98 (**g**–**i**) strains cultured without or with a microsomal fraction (− S9 or + S9) to induce metabolic activation. Values represent the means of the number of revertants (CFU/plate) ± SD (n = 3). ^a^*P* < 0.05, ^b^*P* < 0.01, ^c^*P* < 0.001, ^d^*P* < 0.0001 relative to vehicle-treated control group (Student’s *t*-test).

The strain *S. typhimurium* His^-^ TA102, which is sensitive to a variety of oxidative mutagens^29,30^, was applied to detect mutagenic activity associated with the oxidative potential of ST, its esters (ST-LA and ST-OA), and their thermal degradation products. Tert-butyl-H_2_O_2_ used as a referent oxidant caused a significant response in the TA102 strain as indicated by the mutagenic activity calculated at 7.9 and 2.5, without and with the metabolic activation, respectively (Table 1). The compounds tested (ST, ST-LA, ST-OA) not subjected to the heat treatment did not affect the number of TA102 revertants independently on the microsomal fraction (Fig. 6a–c). After heating, ST and ST-LA could reverse mutation in the TA102 strain. They increased TA102 revertants by approximately 45% (Figs. 5b and 6a). The heated ST-OA did not induce mutation in the TA102 strain, regardless of metabolic activation (+/− S9) (Fig. 6c). However, the low mutagenic index calculated at 1.44, 1.45, and 1.30 for the ST, ST-LA, and ST-OA, respectively, does not provide strong evidence for their mutagenicity.

**Table 1:** Mutagenic index calculated for reference mutagens applied as positive controls to induce mutation in tester strains without or with metabolic activation (−/+ S9).

Reference mutagen	Metabolic activation	Mutagenic index		
		TA98	TA100	TA102
2-Aminofluorene (100 μg)	−	9.78	–	–
Sodium azide (1 μg)	−	–	13.55	–
Tert-butyl-H_2_O_2_ (50 μM)	−	–	–	7.91
2-Aminoanthracene (5 μg)	+	6.00	2.03	2.95
Tert-butyl-H_2_O_2_ (50 μM)	+	–	–	2.51

A positive mutagenic response was observed in the *S. typhimurium* TA100 strain following exposure to the non-heated and heated ST without metabolic activation (Fig. 6d). The mutagenic index values were determined at 1.9 and 2.2 levels, respectively. ST also affected the frequency of mutations in the TA98 strain with microsomal fraction supplementation (Fig. 6g). The number of revertant colonies increased 1.6-fold and 2.5-fold in the TA98 strain treated with non-heated and heated ST, respectively. In contrast, ST esters (ST-LA and ST-OA), regardless of heating treatment, did not induce the reversion of mutations in TA100 and TA98 tester strains in the presence and absence of the microsomal fraction (Fig. 6e,f,h,i).

## Discussion

Functional food enriched in ST and its esters is intended for direct consumption and recommended for cooking, baking, and frying. After thermal treatment, it may constitute a source of thermo-oxidative degradation products, including ST oxidation derivatives and low-molecular-weight compounds, such as volatiles and oligomers^6^, with not well-documented bioavailability, safety, and biological potential to human cells and tissues^19^. Previously reported research showed that thermo-oxidative treatment of ST and its esters (ST-LA and ST-OA) at 180 °C for 8 h causes extensive sterol and fatty acid moiety degradation. Heating produced significant amounts of the ST oxidation products (SOPs) and degradation derivatives, including ST oxides, polar dimers, trimers, other oligomers, and non-polar dimers. ST, ST-OA, and ST-LA degradation products' and SOPs' profiles are shown in the article published previously^25^.

This study assessed cytotoxicity, genotoxicity, and mutagenicity of thermo-oxidatively treated ST, ST-OA, and ST-LA using biological in vitro models. Cytotoxicity analyses: MTT test^25^ and MultiTox-Fluor Multiplex Cytotoxicity Assay indicated the relatively high cytotoxic potential of non-esterified ST. The half-maximal effective concentration (EC_50_) of free ST, which caused a 50% decrease in colon mucosa CCD 841 CoN cell viability, was calculated at 2.95 μg/mL^25^. MultiTox-Fluor Multiplex Cytotoxicity Assay showed that free ST induces a dose-dependent decrease in colon cell viability correlated with an increase in dead cell number. The first cytotoxic effects in the colon cell cultures, reflected in a 10% decrease in the viable cell number, were observed after the treatment with ST in a concentration as low as 1 μg/mL. It has also been shown that free ST at higher doses of 5–40 μg/mL significantly inhibits DNA synthesis in the colon mucosa cells, exerting potent antiproliferative effects. In the literature, ST inhibitory effects were also reported in other human normal cells, including small intestine FHs 74 Int and liver epithelial THLE-2 cells^25^, and human umbilical vein endothelial cells (HUVECs) and iPSC-derived cardiomyocytes^31^. ST was found to affect the viability of cardiovascular-relevant cell models highly. Besides, the harmful cardiac phenotype was observed in stigmasterolemic mice, indicating that ST is a potentially toxic compound^31^.

The experiments performed within this work indicate that ST, independently of thermal treatment, suppressed the proliferation of colon mucosa cells by inhibiting DNA synthesis, arresting the cell cycle in the G_2_/M phases, and targeting cells to the apoptosis pathway. ST anti-proliferative effects were analyzed previously in cancer cell research, which showed that ST can interact with various cellular targets and pathways. For instance, ST has been found to suppress gastric cancer cell proliferation via G_2_/M phase cell cycle arrest and apoptosis induction^32^, consistent with findings on inhibitory effects evoked by ST in normal colon mucosa cells. Thermo-oxidative treatment significantly reduced cytotoxic potential of ST, as observed in the lower potency of heated ST to affect colon cell viability, DNA synthesis, and proliferation. Likewise, the lowered cytotoxicity of the thermally treated ST was noted in previous studies in normal human intestinal and liver cells^25^. The lowering of ST cytotoxicity due to the heating process may be related to the significant degradation of ST molecule, estimated at approximately 80%^25^. However, on the other hand, the thermo-oxidative treatment led to enhanced formation of SOPs, such as 7keto-ST, 7βOH-ST, 7αOH-ST, 25OH-ST, and triol-ST^25^, that show relatively high cytotoxic potential^21^. Among SOPs, 7β-OH-ST, epoxydiol, diepoxide, and triol-ST were identified as the most cytotoxic to the human monocytic U937 cells^21^. The increased SOPs content in ST subjected to the thermo-oxidation process affected the enhanced oxidative reactivity and ST capacity to elevate the intracellular ROS level in treated colon mucosa CCD 841 CoN cells. Excessive ROS accumulation may drive cells into the apoptosis pathway. ROS at low and moderate doses regulate normal physiological functions involved in cell cycle progression and proliferation, cell differentiation, migration, and cell death. While excessive ROS accumulation causes oxidative damage to cellular macromolecules (proteins, lipids, DNA), membranes, and organelles, which may induce apoptotic cell death^33^. The effect of SOPs on intracellular ROS production and ROS-activated apoptosis in normal human cells has not been evidenced previously. However, several studies have suggested a crucial role of oxidative stress in apoptosis induced by COPs^34,35^. In addition, some reports indicated that the cytotoxicity of COPs and POPs is related to superoxide anion generation and lipid peroxidation^36^, the excessive accumulation of which may promote apoptosis^37^.

Apoptosis promotion in the colon mucosa CCD 841 CoN cells treated with ST was manifested by significantly increased caspase 3/7 activity, one of the critical effector caspases involved in the final execution of dying cells. Boosted caspase 3/7 activity was detected in the cells exposed to heated and non-heated ST, although the non-heated ST did not induce enhanced intracellular ROS production. The ST auto-oxidation process and the formation of oxyderivatives, including 7keto-ST and 7βOH-ST^25^, can explain this phenomenon. The SOPs level in unheated ST samples could be too low to enhance ROS generation but high enough to mediate signal transduction pathways. Furthermore, it was found that both non-heated and heated ST caused a slight increase in the cell population with medium DNA damage, which may suggest its potential genotoxicity independent of the thermo-oxidative transformations.

The slight genotoxic effect detected in ST-treated colon mucosa cells was the reason for analyzing the ST mutagenic and pro-mutagenic potential using three mutated *Salmonella typhimurium* TA100, TA102, and TA98 strains. The *Salmonella* tester strains harbor different mutations: hisD3052 (TA98 strain), hisG46 (TA100 strain), and hisG428 (TA102 strain) in the genes of the histidine operon. Cells of the tested strains have also modifications enhancing their sensitivity to mutagenic conditions^30^. The experiments determined the pro-mutagenic activity of tested compounds through their metabolic activation in the presence of Aroclor 1254-induced rat liver microsomal fraction. Non-heated ST showed the ability to reverse mutations in the strain TA100 and strain TA98 only under metabolic activation. However, the mutagenic indexes not exceeding 2.0 in values, as required for mutagenic compounds, did not indicate ST mutagenic potential. For comparison, the mutagenic index of 2-aminofluorene and sodium azide, applied as reference mutagens for TA98 and TA100 strains, was determined at 9.8 and 13.6, respectively. Therefore, the non-heated ST was not considered mutagenic. More significant effects were detected in the *Salmonella* strains induced by ST subjected to thermo-oxidative treatment. Interestingly, the heated ST increased the number of revertants in the TA102 strain sensitive to various oxidative mutagens^30^, indicating the pro-oxidative capacity of ST derivatives produced during the thermo-oxidative process. SOPs generated when ST was heated at 180 °C were likely responsible for the oxidative mutagenicity detected in the TA102 strain. In contrast, the mutation reversal was not observed in the TA102 strain treated by heated ST under conditions stimulating metabolic bioconversions. These findings indicate that natural metabolic and antioxidant systems may limit the mutagenic activity of oxidant SOPs during the thermal proceedings. Moreover, considering the low mutagenic index (< 2.0), the oxidative mutation risk is probably relatively low regardless of metabolic activation. However, a significant increase in the mutation frequency was observed in the TA98 strain treated with heated ST under metabolic activation. In this experiment, the mutagenic index was determined at 2.5, indicating the potential pro-mutagenicity of ST after thermal treatment and metabolic transformation.

Previous studies have shown that free ST is degraded rapidly, generating a diverse group of thermo-oxidative degradation products and oxyderivatives^21,25^ which may exhibit significant oxidative reactivity and induce cytotoxic and genotoxic effects in normal human cells as demonstrated in the presented studies. It was found that ST esterification with oleic and linoleic acids limited ST degradation and the formation of oxidized sterols, degradation products, and oligomers^25,38^, leading to a reduction in the risk of adverse toxic effects. Cytotoxicity experiments proved the lower cytotoxic potential of stigmasteryl esters (ST-OA and ST-LA) than free ST, regardless of whether they were subjected to thermo-oxidative treatment. Unlike ST, non-heated ST-OA and ST-LA at concentrations up to 40 μg/mL were not oxidatively reactive, and they did not affect the viability and proliferation of normal human colon cells. Similarly, no cytotoxic effects of ST-LA and ST-OA esters were observed in normal human small intestine FHs 74 Int cells and liver THLE-2 cells^25^. However, the small apoptotic cell populations with slightly increased caspase-3/7 activities were identified in the colon cell cultures treated with ST-LA and ST-OA at the maximum dose tested. Unfortunately, the thermo-oxidative process caused ST-LA and ST-OA degradation, including sterol moiety and fatty acid residue, and ST oxyderivatives production, thereby altering their biological activity. But, it should be pointed out that the extent of ST-LA and ST-OA ester degradation and SOPs production was much lower than that documented for non-esterified ST. For instance, after heating, the total SOPs content in the ST-OA and ST-LA was 4.3- and 1.9- fold lower than in ST^25^. After thermo-oxidative treatment, ST-LA cytotoxicity and oxidative reactivity were significantly increased accordingly to the thermo-oxidative degradation extent and content of oxysterols, which elevated 8.4-fold under treatment. In contrast to ST-LA, the heating process did not enhance ST-OA cytotoxicity and oxidative reactivity. The heated ST-OA did not induce cytotoxic and genotoxic effects nor elevate the intracellular ROS level in normal human colon cells. Similarly, the different cytotoxic potency of thermally treated ST-LA and ST-OA was also observed in human acute lymphoblastic leukemia cell cultures^38^. The higher ST-LA reactivity was probably due to the 2.3-fold higher content of SOPs^25^. Elevated ROS levels in the colon mucosa cells after exposure to heated ST-LA suggest the SOPs' involvement in cytotoxic and pro-oxidative effects. Intracellular ROS overproduction likely mediated the initiation of the apoptosis pathway with the increase in caspase 3/7 activity. However, no significant oxidative DNA strand breaks were detected in the cells treated with heated ST-LA. Although, SOPs generated under ST-LA thermo-oxidative processing resulted in a 45% increase in the mutation frequency in the highly oxidant-sensitive TA102 strain. Nevertheless, in the presence of the microsomal S9 fraction used for metabolic activation, mutations were not induced in the TA102 strain by thermo-oxidative ST-LA derivatives. A similar detoxifying effect of metabolic activation was also observed in the TA102 strain incubated with the heated non-esterified ST. Antioxidant enzymes such as catalase or superoxide dismutase, contained in S9 liver fraction^39^, may mitigate ROS generated by oxyderivatives. In contrast to heated ST-LA, heated ST-OA did not increase mutation frequency in the TA102 strain independently on metabolic activation. A few studies on ST, its esters, and SOPs mutagenicity have been performed to date. Phytosterol preparations containing a well-defined ST contribution, but not individual ST compounds, were analyzed^3^. The Salmonella reverse mutation assay showed no mutagenicity of the mixture of phytosterol esters with stigmasteryl esters contribution at 18% in the strains TA98, TA100, TA1535, and TA1537 with and without metabolic activation^40^. Also, the phytosterol oxide mixture formed by prolonged heating of phytosterol ester concentrate containing ST ester (19%) did not have the mutagenic potential in bacterial strains TA98, TA100, TA1535, TA1537, and TA102^41^. Most scientific reports indicate no severe side effects of phytosterols, including ST, on human health. However, according to scientists, toxicological studies should be continued to provide evidence of the safety and health-promoting effects of ST, functionally modified ST, ST-enriched functional foods, and derivatives generated during the storage and heating process.

## Conclusions

This study showed the significant cytotoxic effects of free ST on normal human colon mucosa cells by suppressing cell proliferation, inhibiting DNA synthesis, arresting the cell cycle in the G_2_/M phases, and targeting cells to the apoptosis pathway. The high ST oxidative reactivity was also manifested by intracellular ROS accumulation after intestinal cell treatment. The cytotoxicity-reducing effect was obtained by ST esterifying with oleic and linoleic acids. Upon ST esterification, ST-LA and ST-OA at doses up to 40 μg/mL did not induce cytotoxic effects or inhibit normal colon cell proliferation. The findings indicate the higher toxicological safety of ST incorporated into the ester molecule than free ST.

Previous studies have shown that the esterification of ST with oleic and linoleic acids impedes the ST degradation and formation of oxidized sterols, degradation products, and oligomers during thermo-oxidation treatment^25^. Limiting the formation of thermo-oxidative ST derivatives—compounds of unknown bioavailability and non-defined impacts on human cells and tissues is an important issue for maintaining the safety of ST-enriched food products, especially during cooking and processing. ST-LA and ST-OA stigmasteryl esters differ in forming degradation products and oxyderivatives during thermo-oxidative treatment and, thus, also in cytotoxicity and oxidative reactivity. ST-OA is characterized by higher toxicological safety; independently of thermal treatment, it does not induce cytotoxic and genotoxic effects nor elevate the intracellular ROS level in normal human colon cells. In contrast to ST-OA, the heating causes enhanced ST-LA oxidative reactivity and increases its cytotoxic potential in the intestinal cells. The findings suggest that ST-OA may constitute a non-cytotoxic ST compound to form special-purpose functional foods intended for direct consumption and the thermal proceeding. However, further preclinical studies should be continued to determine ST-OA bioavailability and functionality and exclude unfavorable health effects, such as pro-atherogenicity or pro-inflammatory.

## Materials and methods

### Preparation of stigmasteryl esters

Stigmasterol—ST (≥ 95%), oleic acid—OA (≥ 99%), and linoleic acid—LA (≥ 99%) standards were purchased from Sigma-Aldrich (St. Louis, MO, USA). ST-OA and ST-LA were obtained by chemical esterification based on the Neises and Steglich method^42^ according to the protocol described by Kasprzak et al.^25^. ST (500 mg) dissolved in dichloromethane (30 mL) was placed in a three-necked flask. The air in the flask was replaced with argon. The catalyst: N,N′-Dicyclohexylcarbodiimide (500 mg) and 4-Dimethylaminopyridine (15 mg), and fatty acid (OA or LA) (600 mg) were then added to the flask. Esterification was performed at room temperature for 24 h in the dark. The reaction mixture was transferred to a separatory funnel and extracted in triplicate with distilled water (10 mL), with lower layers collecting. The fractions collected were concentrated under a vacuum at 30 °C, and the residue was dissolved in hexane (20 mL). The esterification mixture was purified on a silica gel column (45 × 2.5 cm). The ester fraction was eluted with hexane:ethyl acetate (9:1) mixture (450 mL). TLC was used to check the ester fraction purity.

### Thermo-oxidative treatment

ST, ST-OA, and ST-LA (50 mg each) were placed separately in glass vials and heated at 180 °C for 8 h under an oxygen atmosphere. After thermo-oxidative treatment, the tested compounds were stored at − 20 °C until chemical and biological analyses. The non-heated and heated ST and its esters were analyzed to detect degradation products and oxyderivatives. Analytical methods were described in the article published previously^25^. Table S1 presents the composition of thermo-oxidative degradation products of ST, ST-OA, and ST-LA.

### Compounds preparation for cytotoxicity and genotoxicity experiments

ST, ST-LA, and ST-OA were dissolved in acetone and then diluted in this solvent to obtain the concentrated stock solutions of the analyzed compounds at each dose tested. Each concentration was prepared by 200-fold diluting the appropriate stock solution in a culture medium.

### Cytotoxicity assay

The normal human diploid CCD 841 CoN cell line (ATCC^®^ CRL-1790™) isolated from colon mucosa was obtained from American Type Culture Collection (ATCC, Manassas, VA, USA). Cells were cultured in Dulbecco’s Modified Eagle’s Medium (DMEM, Sigma-Aldrich) supplemented with fetal bovine serum (FBS; Gibco BRL, Grand Island, NY, USA) to a final concentration of 10% and maintained at 37 °C in a humidified atmosphere with 5% CO_2_.

In the cytotoxicity experiments, CCD 841 CoN cells were seeded in 96-well plates at an initial density of 2.0 × 10^4^ cells/cm^2^ and incubated for 24 h. Then, the cells were exposed to the non-heated and heated ST, ST-OA, and ST-LA at concentrations of 1.25, 2.5, 5, 10, 20, and 40 μg/mL for 48 h under standard culture conditions. Control culture contained the vehicle at the amount corresponding to the sample analyzed.

The Multitox-Fluor Multiplex Cytotoxicity Assay (Promega GmbH, Mannheim, Germany) was applied to determine the relative number of live and dead cells in the CCD 841 CoN cell cultures treated with the analyzed compounds. The assay determined live- and dead-cell protease activities using two fluorogenic peptide substrates, cell-permeant (glycyl-phenylalanyl-amino fluorocoumarin; GF-AFC) and cell-impermeant (bis-alanyl-alanyl-phenylalanyl-rhodamine 110; bis-AAF-R110). After treatment, the cells were incubated with GF-AFC and bis-AAF-R110 substrates for 60 min at 37 °C. The live- and dead-cell proteases produced AFC and R110 products, measured using a Tecan M200 Infinite microplate reader (Tecan Group Ltd., Männedorf, Switzerland) at different excitation (400 nm and 485 nm) and emission (505 nm and 520 nm) spectra. The assay was conducted according to the manufacturer’s instructions.

### Measurement of DNA synthesis

CCD 841 CoN cells were grown in black 96-well plates at an initial cell density of 2 × 10^4^ cells/cm^2^ for 24 h under standard culture conditions. The cells were treated with ST (2.5, 5, 10, 20, and 40 μg/mL) and its esters ST-OA and ST-LA (10, 20, and 40 μg/mL) for 48 h. DNA synthesis was measured by incorporating thymidine analog 5-bromo-2′-deoxyuridine (BrdU) into newly synthesized DNA using the Cell Proliferation ELISA kit according to the protocol recommended by the manufacturer (Roche Diagnostics GmbH, Mannheim, Germany). Briefly, BrdU was added to the treated cells 24 h before the end of the exposure to the ST, ST-OA, and ST-LA (final BrdU concentration of 10 μM). After treatment, BrdU-labeled DNA was denatured (30 min, 20 °C). Then, BrdU was bonded with a peroxidase-conjugated anti-BrdU antibody (90 min, 20 °C) and reacted with the peroxidase substrate. The absorbance measurement was done with a stop solution (1 M H_2_SO_4_) at 450 nm with reference wavelength 690 nm using a Tecan M200 Infinite microplate reader.

### Cell cycle analysis

CCD 841 CoN cells were grown in 6-well plates at an initial cell density of 2 × 10^4^ cells/cm^2^ for 24 h under standard culture conditions. The cells were treated for 24 h with the analyzed compounds at a concentration of 40 μg/mL; the high dose was applied because of the relatively low cytotoxic potential of ST-OA and ST-LA as determined in cytotoxicity studies. In addition, the cells were exposed to 0.05 μM camptothecin (Sigma-Aldrich) as a positive reference compound known to modulate cell cycle progression.

After treatment, the cells were harvested by trypsinization, washed in PBS, and fixed in 70% ethanol. Then, the cells were stained with 50 μg/mL propidium iodide in the presence of 100 μg/mL RNase (Sigma-Aldrich). The sample preparation method and staining protocol were described previously^43^. The cycle phase distribution was analyzed with an Amnis™ FlowSight™ flow cytometer (Luminex Corporation, TX, USA).

### Caspase 3/7 activity assay

CCD 841 CoN cells were grown in black 96-well plates at an initial cell density of 2 × 10^4^ cells/cm^2^ for 24 h under standard culture conditions. The cell cultures were treated for 24 h with ST, ST-OA, and ST-LA at a 40 μg/mL dose. After treatment, caspase-3/7 activity was determined using the APO-One Homogeneous Caspase-3/7 Assay (Promega Corporation, Wisconsin, USA), which is based on the proteolytic cleavage of C-terminal side of aspartate residue in the DEVD peptide substrate by caspase 3/7 into fluorescent rhodamine 110 (R110). Analysis of caspase 3/7 activity was carried out following the manufacturer's instructions. Briefly, the cells after the treatment were incubated at room temperature with Apo-ONE^®^ Caspase-3/7 Reagent—bifunctional cell lysis/caspase activity buffer combined with the pro-fluorescent caspase-3/7 substrate Z-DEVD-R110. After 4-h incubation, fluorescence was measured at an excitation wavelength of 485 nm and an emission wavelength of 530 nm using a Tecan M200 Infinite microplate reader. Data obtained were normalized to cellular protein content, quantified by BCA assay (Pierce^®^ BCA Protein Assay Kit, Thermo Scientific Inc., USA) according to the manufacturer's protocol.

### Intracellular ROS measurement

CCD 841 CoN cells were grown in 6-well plates at an initial cell density of 2 × 10^4^ cells/cm^2^ for 24 h under standard culture conditions. The cell cultures were treated for 24 h with the analyzed compounds at a 40 μg/mL concentration. After treatment, the cells were harvested by trypsinization, washed with Hank's Balanced Salt Solution, and incubated with 10 μM 2′,7′-Dichlorodihydrofluorescein diacetate (DCFH-DA) (Life Technologies, Carlsbad, CA, USA) at 37 °C for 30 min. Intracellular DCF fluorescence (λex/em = 488/530 nm) was examined by flow cytometry (Amnis™ FlowSight™ flow cytometer).

### DNA damage detection

The cells were grown at 6-well plates at the established density and standard culture conditions and exposed to the non-heated and heated ST, ST-OA, and ST-LA for 48 h. The non-treated cells and cells treated with H_2_O_2_ (100 μM, 30 min) to induce oxidative DNA damage constituted the negative and positive controls, respectively. After treatment, cells were analyzed for DNA damage using a single cell gel electrophoresis (SCGE) called comet assay, described in detail previously^44^. Briefly, harvested cells suspended in low melting point agarose were put onto microscope slides pre-coated with normal melting point agarose and subjected sequentially to lysis, alkaline electrophoresis (pH > 13), and neutralization (pH 10). The cells were stained with SYBRGold (Molecular Probes), viewed under a fluorescence microscope (Axiovert 200, Zeiss, Carl Zeiss, Gottingen, Germany), and analyzed using CometScore™ software (TriTek Corp., Sumerduck, VA, USA). At least 100 cells were analyzed on a microscope slide; 300 cells were considered for DNA damage detection in one sample. Data were expressed as the mean percentage of DNA content in the comet tail. Moreover, depending on DNA content in comet tails, comets were classified into five categories: class 0 (< 1%, no damage), class 1 (1–25%; low damage), class 2 (> 25–45%; medium damage), class 3 (> 45–70%; high damage), class 4 (> 70%; very high damage). Based on the comets' categorization, the total comet score (TCS) was calculated according to the formula: TCS = 0(n) + 1(n) + 2(n) + 3(n) + 4(n), where “n” is the number of cells in each comet class (0–4)^45^.

### Mutagenicity assay

Potential mutagenicity or pro-mutagenicity of non-heated and heated ST, ST-LA, and ST-OA was determined using the bacterial reverse mutation assay (Ames test) with *Salmonella enterica* subsp. *enterica* ser. *typhimurium* tester strains TA98, TA100, and TA102, which were obtained from the Polish Collection of Microorganisms of the Institute of Immunology and Experimental Therapy of the Polish Academy of Sciences in Wroclaw.

Mutagenicity experiments were performed in the liquid pre-incubation assay, without and with metabolic activation by supplying Aroclor 1254-induced rat liver microsomal fraction (S9, Sigma-Aldrich) with NADP and cofactors for NADPH-supported oxidation, according to the procedure described by Mortelmans and Zeiger^39^. Briefly, reaction mixtures, consisting of 12-h bacterial culture, non-heated and heated compound (ST, ST-LA, ST-OA) at a concentration of 40 μg, 0.2 M phosphate buffer (pH 7.4), and alternatively S9 activating mixture, were pre-incubated at 37 °C for 20 min. The mutagens, 2-aminofluorene (100 μg), sodium azide (1 μg), and tert-butyl-hydrogen peroxide (tert-butyl-H_2_O_2_) (50 μM) (all supplied by Sigma-Aldrich), were used as positive controls to reverse mutation in the TA98, TA100, and TA102 strain, respectively. The mutagen, 2-aminoanthracene (5 μg) (Sigma-Aldrich), was a positive control in pro-mutagenicity experiments. After pre-incubation, the reaction mixtures were added to the top agar supplemented with traces of histidine and biotin and poured onto the plates covered with minimum glucose agar. The cultures were incubated for 48 h at 37 °C, and the number of revertants (His+) colonies was counted manually.

The mutagenic index (MI), which reflects the number of induced revertants (Ri) with mutagen or compound tested divided by the number of spontaneously induced revertants (Rs), was applied to express mutagenic activity. Compounds with MI ≥ 2 can be recognized as potentially mutagenic^46^. The MI values calculated for reference mutagens applied to induce reverse mutation in tester strains ranged from 2.51 to 13.55 (Table 1).

### Statistical analysis

Data are presented as means ± SD from three independent replications. Statistical analysis was performed using STATISTICA version 13.3 software (Statsoft, Inc., Tulsa, OK, USA). A Student’s *t*-test was used to compare two groups of data. One-way analysis of variance (ANOVA) followed by Tukey’s post hoc test was performed to determine the differences between the mean values of multiple groups. *P* ≤ 0.05 was the cut-off point for a significant difference.

## Supplementary Information

Supplementary Table S1.

## Data Availability

The datasets generated during and/or analyzed during the current study are available from the corresponding author on reasonable request.
