# Supplementary material for: Anti-proliferative potential and oxidative reactivity of thermo-oxidative degradation products of stigmasterol and stigmasteryl esters for human intestinal cells
Source: Sci Rep. 2023 May 1;13:7093. doi: 10.1038/s41598-023-34335-0 (PMC10151334; doi:10.1038/s41598-023-34335-0)
Supplement: Supplementary file 1 — Supplementary Table S1. [file 41598_2023_34335_MOESM1_ESM.pdf]

Tab.S1 Thermo-oxidative degradation products and oxidation derivatives formed during thermal treatment of stigmasterol (ST) and stigmasteryl oleate (ST-OA) and linoleate (ST-LA) at 180 °C for 8 h. The compound content was calculated for the maximum dose (40 μg/mL) used in the cytotoxicity and genotoxicity experiments.

**Fraction**	**Compounds**	**Stigmasterol (ST)**		**Stigmasteryl oleate (ST-OA)**		**Stigmasteryl linoleate (ST-LA)**	
		Unheated	Heated	Unheated	Heated	Unheated	Heated
Polar	Fragmented[#]	0.01 ± 0.00	0.70 ± 0.01	0.01 ± 0.00	0.65 ± 0.05	0.01 ± 0.00	0.57 ± 0.04
(μg/40 μg/mL)	Monomers[*]	0.12 ± 0.00	9.27 ± 0.21	0.19 ± 0.02	9.46 ± 0.12	0.19 ± 0.01	6.07 ± 0.11
	Dimers	Nd	2.68 ± 0.06	Nd	0.83 ± 0.06	Nd	2.51 ± 0.07
	Trimers	Nd	1.46 ± 0.11	Nd	0.50 ± 0.04	Nd	0.57 ± 0.01
	Other oligomers	Nd	Nd	Nd	Nd	Nd	0.24 ± 0.02
	Total oligomers	Nd	4.14 ± 0.13	Nd	1.33 ± 0.10	Nd	3.33 ± 0.04
Non-polar	Fragmented[#]	Nd	0.39 ± 0.03	1.78 ± 0.03	4.85 ± 0.07	1.10 ± 0.04	10.77 ± 0.12
(μg/40 μg/mL)	Monomers[*]	39.88 ± 0.09	15.22 ± 0.06	38.02 ± 0.10	23.63 ± 0.14	38.70 ± 0.17	19.19 ± 0.10
	Dimers	Nd	10.27 ± 0.10	Nd	0.08 ± 0.00	Nd	0.08 ± 0.00
Total oligomers (μg/40 μg/mL)		Nd	14.42 ± 0.23	Nd	1.41 ± 0.10	Nd	3.41 ± 0.12
Total SOPs (ng/40 μg/mL)		9.71 ± 0.24	97.23 ± 4.38	4.21 ± 0.11	22.42 ± 1.28	6.06 ± 1.40	51.08 ± 2.76
7α-hydroxy-ST		2.15 ± 0.15	10.90 ± 0.90	0.59 ± 0.03	6.35 ± 0.94	2.00 ± 0.13	10.59 ± 0.18
7β-hydroxy-ST		2.22 ± 0.22	26.51 ± 0.59	0.35 ± 0.00	2.34 ± 0.07	0.90 ± 0.13	20.81 ± 1.97
5β,6β-epoxy-ST		Nd	2.48 ± 0.03	0.78 ± 0.10	2.54 ± 0.03	0.65 ± 0.11	5.12 ± 0.39
5α,6α-epoxy-ST		2.59 ± 0.08	2.60 ± 0.05	0.26 ± 0.06	4.25 ± 0.53	0.40 ± 0.04	5.53 ± 0.01
Stigmasten-3β,5α,6β-triol		Nd	10.20 ± 1.90	Nd	2.78 ± 0.08	0.12 ± 0.01	2.65 ± 0.13
25-hydroxy-ST		1.48 ± 0.27	16.98 ± 0.47	0.82 ± 0.00	1.46 ± 0.04	0.41 ± 0.00	2.58 ± 0.05
7keto-ST		1.27 ± 0.12	27.57 ± 0.44	1.42 ± 0.02	2.70 ± 0.12	1.57 ± 1.05	3.80 ± 0.13
Sterol moiety depletion (%)		-	↓80.4 ± 13.5	-	↓88.8 ± 3.5	-	↓71.6 ± 6.6
Fatty acid residue depletion (%)		-	-	-	↓77.14 ± 5.3	-	↓31.11 ± 3.2

[#]fragmented – partial ST, ST-OA, and ST-LA molecules; [*]monomers - ST, ST-OA, and ST-LA non-degraded molecules
